# Construction of a lncRNA-mediated feed-forward loop network reveals global topological features and prognostic motifs in human cancers

**DOI:** 10.18632/oncotarget.10004

**Published:** 2016-06-14

**Authors:** Shangwei Ning, Yue Gao, Peng Wang, Xiang Li, Hui Zhi, Yan Zhang, Yue Liu, Jizhou Zhang, Maoni Guo, Dong Han, Xia Li

**Affiliations:** ^1^ College of Bioinformatics Science and Technology, Harbin Medical University, Harbin, 150081, China; ^2^ National Center for Nanoscience and Technology, Beijing, 100190, China

**Keywords:** long non-coding RNA, feed-forward loop, network motif, topological feature, prognostic biomarker

## Abstract

Long non-coding RNAs (lncRNAs), transcription factors and microRNAs can form lncRNA-mediated feed-forward loops (L-FFLs), which are functional network motifs that regulate a wide range of biological processes, such as development and carcinogenesis. However, L-FFL network motifs have not been systematically identified, and their roles in human cancers are largely unknown. In this study, we computationally integrated data from multiple sources to construct a global L-FFL network for six types of human cancer and characterized the topological features of the network. Our approach revealed several dysregulated L-FFL motifs common across different cancers or specific to particular cancers. We also found that L-FFL motifs can take part in other types of regulatory networks, such as mRNA-mediated FFLs and ceRNA networks, and form the more complex networks in human cancers. In addition, survival analyses further indicated that L-FFL motifs could potentially serve as prognostic biomarkers. Collectively, this study elucidated the roles of L-FFL motifs in human cancers, which could be beneficial for understanding cancer pathogenesis and treatment.

## INTRODUCTION

Long non-coding RNAs (lncRNAs, > 200 nucleotides in length) [1] are pervasive across the genome [2, 3] and dysregulation of their expression is associated with many human diseases [4, 5], including cancer [6]. The expression of lncRNA is regulated at the transcriptional level by transcription factors (TFs) [7] and at the post-transcriptional level by microRNAs (miRNAs) [8–10], leading to differential expression in different development and disease statuses. While both TFs and miRNAs regulate lncRNAs, TFs also regulate miRNAs [7]. This lncRNA-mediated feed-forward loop (L-FFL) is an important regulatory network motif underlying many biological processes, such as muscle cell differentiation [11] and cancer [12]. Indeed, many lncRNAs, TFs and miRNAs are dysregulated in various types of cancer [13, 14]. However, there has been no large-scale attempt to identify L-FFL network motifs and their specific roles in human cancers.

Large-scale cancer genomics projects, such as the Cancer Genome Atlas (TCGA), have provided the global expression profiles of some lncRNAs, TFs and miRNAs in large samples [15, 16]. In addition, several databases have been developed to facilitate the construction of L-FFL network motifs. For example, SNP@lincTFBS and ChiPBase identify TF-lncRNA interactions from ChIP-Seq data [17, 18]. DIANA-LncBase and starBase v.2.0 provide experimentally verified miRNA-lncRNA interactions from Ago CLIP-seq data [19, 20]. TransmiR manually collects experimentally supported TF-miRNA regulatory relationships from literature and publications [21]. However, these databases provide limited insight into the structure and function of the L-FFL network.

We hypothesized that the integration of large-scale cancer expression profiling data and an L-FFL network to identify cancer-associated L-FFL motifs may reveal novel regulatory mechanisms in human cancers as well as potential therapeutic targets. For this study, we developed a computational approach that integrates interaction and expression data of lncRNAs, TFs and miRNAs to identity dysregulated L-FFL motifs common across and specific to six types of human cancer. Survival analyses suggest that L-FFL motifs may could potentially serve as prognostic biomarkers.

## RESULTS

### The topological characteristics of the L-FFL network

**Figure 1:**
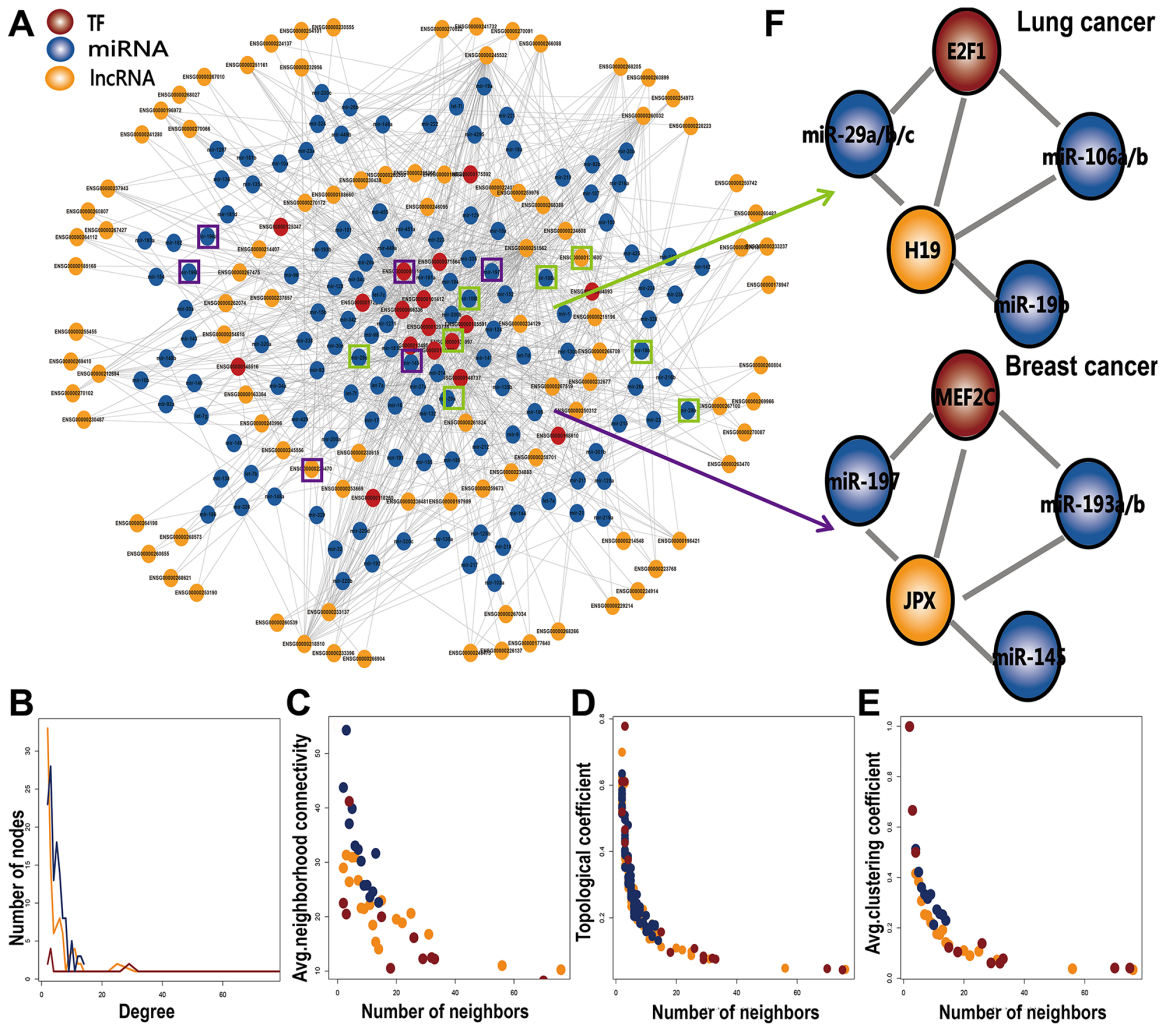
The basic characteristics of L-FFL network **A.** A global L-FFL network. lncRNAs, miRNAs and TFs are colored yellow, blue and red, respectively. Known disease-associated nodes are marked by black circles. **B–E.** The basic features of the network include degree, connectivity, topological coefficients, and clustering coefficients of lncRNA, miRNA and TF. **F**. Two L-FFL motifs were associated with lung cancer and breast cancer.

We integrated multiple data sources to identify 623 L-FFL network motifs, each consisting of a TF, an miRNA and their common target lncRNA and then constructed a global L-FFL network containing 240 nodes (96 lncRNAs, 17 TFs and 127 miRNAs) and 878 edges (Figure 1A). We found that this network approximated the scale-free network topology of a transcriptional regulatory network. The TFs held a larger degree than the lncRNAs and miRNAs and participated in more regulatory relationships (Figure 1B). We also analyzed connectivity, topological coefficient, and clustering coefficient of nodes. All of these features followed a scale-free distribution (Figures 1C, 1D, 1E), indicating that the L-FFL network behaves like a small-world phenomenon [22]. The neighborhood connectivity distribution provides the average of the neighborhood connectivity of lncRNAs, TFs, and miRNAs with k neighbors (k =0, 1…n). The constant decrease in the topological coefficient as the degree increases per lncRNA, TF, and miRNA indicates that the networks may have a hierarchical modularity. If the distribution decreases, then most of the edges in the network connect low-degree nodes with high-degree nodes, indicating that the network consists of sub-networks [23]. Some other networks have similar topological features. For example, the ceRNA network in cancer usually shows the features of multiple layers and being scale-free [24].

In addition, some nodes and edges in the L-FFL network were found to be associated with human cancer in previous studies. We identified six lncRNAs, 17 TFs and 127 miRNAs that were associated with different types of human cancers using data from lncRNADisease [25], HMDD [26] and the literature (Figure 1A, Supplementary Table S1). For example, the lncRNA JPX, the TFs MYC and E2F1 and the miRNA let-7 play crucial roles in human cancers [27, 28]. More importantly, we found that some L-FFL motifs provide novel information on cancer regulation. For example, MYC is an onco-protein family comprised of c-myc, N-myc and L-myc, all of which contribute to pathogenesis in many human cancers. The lncRNA H19, which is regulated by c-myc, participates in embryonic development and tumorigenesis. Upregulation of H19 promotes cell proliferation correlates with poor prognosis in non-small-cell lung cancer [29]. MiR-29a/b/c, miR-106a/b and miR-19b are known disease-related miRNAs, especially in lung cancer [30–32]. Our L-FFL network is composed of L-FFL motifs in lung cancer, which may represent a new mechanism in cancer (Figure 1F). As another example, the lncRNA JPX is an Xist activator, yet Xist expression is downregulated in breast cancer [33]. The TF MEF2C is expressed in normal mammary epithelial cells and in breast cancer cell lines [34]. MiR-193a/b, miR-145, and miR-197 were previously reported to promote breast cancer [35–37]. The TF MEF2C interacts with the lncRNA JPX, miR-193a/b, miR-145, and miR-197, and every transcript in these structures is associated with breast cancer (Figure 1F), suggesting they may play critical roles in tumorigenesis in combination with an L-FFL motif.

### Some L-FFL motifs are significantly dysregulated in cancer

**Figure 2:**
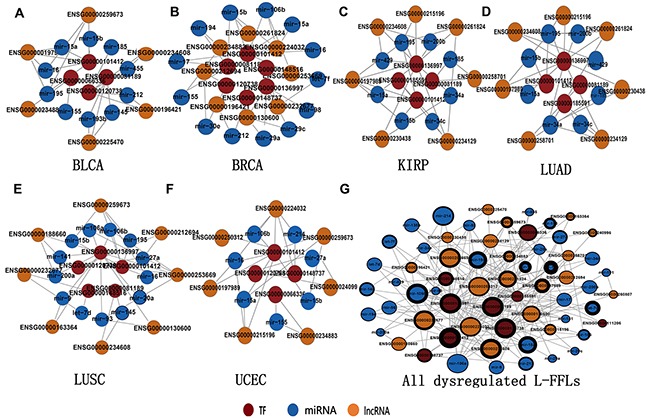
The sub-network of significantly dysregulated L-FFL motifs A significantly dysregulated sub-network related to **A.** BLCA, **B.** BRCA, **C.** KIRP, **D.** LUAD, **E.** LUSC and **F.** UCEC, and **G.** the union network of six sub-networks. LncRNAs, miRNAs and TFs are colored yellow, blue and red, respectively. The node size represents the degree of the node in the network, and the thickness of the node border represents the number of cancers in which the nodes participated.

To further understand the functional significance of L-FFL motifs in human cancer, we identified the significantly dysregulated L-FFL network motifs for different cancer types in the L-FFL network (see Materials and Methods). Based on these motifs, we constructed significantly dysregulated L-FFL sub-networks for each cancer type (Figure 2, Supplementary Table S2). There were 12 L-FFL motifs with five lncRNAs, four TFs and 10 miRNAs in bladder cancer (Figure 2A); 16 L-FFL motifs with eight lncRNAs, six TFs and 13 miRNAs in breast cancer (Figure 2B); 14 L-FFL motifs with seven lncRNAs, four TFs and eight miRNAs in kidney cancer (Figure 2C); 11 L-FFL motifs with seven lncRNAs, five TFs and eight miRNAs in lung adenocarcinoma (Figure 2D); 15 L-FFL motifs with eight lncRNAs, five TFs, and 13 miRNAs in lung squamous cell carcinoma (Figure 2E); and 12 L-FFL motifs with seven lncRNAs, four TFs and seven miRNAs in uterine corpus endometrioid carcinoma (Figure 2F).

Analyzing these sub-networks, we found that some nodes and edges were present in multiple types of cancer, suggesting they play important roles in human carcinogenesis. To investigate these nodes, we first constructed a highly dysregulated L-FFL sub-network consisting of all of the dysregulated motifs for all six types of cancer. This network included 21 lncRNAs, nine TFs and 34 miRNAs (Figure 2G). The nodes with larger degrees were usually associated with more types of cancer and tended to be network hubs, indicating they might promote pan-cancer development. For example, the TF E2F1, the lncRNA H19 and the miRNA miR-106 had high degrees and were dysregulated in many types of cancer. Indeed, these transcripts are known to promote cancer development [38, 39]. Furthermore, we performed an enrichment analysis to investigate the function of this sub-network using all of the TFs [40]. These TFs were enriched in several cancer-related GO terms and pathways, such as the cancer pathway (Supplementary Figure S1). Significant GO terms included transcriptional regulation, transcription factor activity and positive regulation of RNA metabolic processes (Supplementary Table S3).

### Common and specific dysregulated L-FFL motifs across cancer types

**Figure 3:**
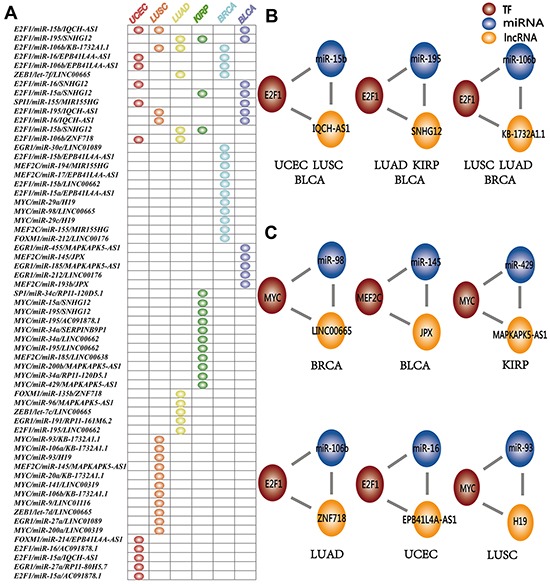
Common and specific dysregulated L-FFL motifs **A.** L-FFL motifs dysregulated across cancer types. Each row represents an L-FFL motif, and each column represents one type of cancer. The node in the table indicates that this L-FFL motif was dysregulated in this cancer. UCEC, LUSC, LUAD, KIRP, BRCA and BLCA are red, orange, yellow, green, blue and purple, respectively. **B.** Three examples of common L-FFL motifs that were dysregulated in at least three types of cancer. **C.** Six examples of specific L-FFL motifs that were dysregulated in BRCA, BLCA, KIRP, LUAD, UCEC and LUSC.

Here, L-FFL motifs dysregulated in at least two cancer types are designated as “common”, while those dysregulated in only one cancer type are considered “specific”. In total, 48 L-FFL motifs were identified as cancer specific, and 13 L-FFL motifs were common between different cancer types (Figure 3A). Some common L-FFL motifs were dysregulated in at least three types of cancer (Figure 3B, Supplementary Table S4). For example, an L-FFL motif consisting of the TF E2F1, the miRNA miR-15b and the lncRNA IQCH-AS1 was dysregulated in breast cancer, lung squamous cell carcinomas, and lung adenocarcinoma. Previous studies have demonstrated that E2F1 can regulate the expression of genes in the cell cycle and act as a tumor suppressor in many types of cancer [41]. Also, miR-15b plays a critical role in many types of cancer and miR-15 families known as oncomiRs have tumor suppressor or oncogene functions [42]. We found that the lncRNA IQCH-AS1 interacts with E2F1 and miR-15b, suggesting it might be implicated in cancer regulation through the L-FFL network. Thus, the L-FFL might underlie cancer development processes common to many types of cancer. In addition, we also found that the L-FFL motif comprised of E2F1, miR-195 and SNHG12, and the L-FFL motif comprised of E2F1, miR-106b and KB-1732A1.1, were dysregulated in three types of cancers (Figure 3B). E2F1 appeared many times in common L-FFL motifs and therefore might play different roles in different types of cancers by interacting with different lncRNAs and miRNAs [38]. On the other hand, most motifs were specific (Figure 3C, Supplementary Table S4). For example, the MYC, miR-98 and LINC00665 motif was dysregulated only in breast cancer, while the MEF2C, miR-145 and JPX motif was dysregulated only in bladder cancer.

### L-FFL motifs as prognostic biomarkers for cancers

**Figure 4:**
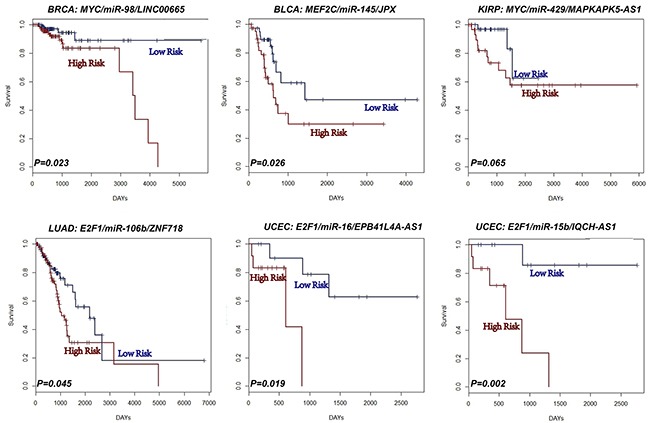
L-FFL motifs are potential prognostic biomarkers for cancers Kaplan-Meier survival analysis performed on two groups of patients with different clinical outcomes. The blue lines represent the group with low risk, and the red lines represent the group with high risk.

We tested whether the expression of each L-FFL motif correlated with cancer survival (see Materials and Methods). Indeed, the expression of some L-FFL motifs correlated with survival in all types of cancer tested, excluding lung squamous cell carcinomas (Figure 4, Supplementary Table S5). For example, the L-FFL motif comprised of MYC, miR-98 and LINC00665 correlated with breast cancer patient survival (P=0.023), in agreement with previous studies showing that MYC and miR-98 correlate with cancer patient survival [43, 44]. Also, the MEF2C, miR-145 and JPX correlated with the survival of bladder cancer patients (P=0.026). In addition, the MYC, miR-429 and MAPKAPK5-AS1 motif correlated with kidney cancer patient survival (P=0.065); the E2F1, miR-106b and ZNF718 motif correlated with lung adenocarcinoma patient survival (P=0.045); and, finally, two motifs correlated with endometrial cancer patient survival (P=0.019 and P=0.002). Importantly, survival analyses using only one type of transcript had no prognostic power. For example, the MYC, miR-429 and MAPKAPK5-AS1 motif did not correlate with patient survival when analyzing each of its components separately. These results suggest that L-FFL motifs may serve as prognostic biomarkers for different cancers.

### L-FFL motifs may participate in complex biologic network regulation

**Figure 5:**
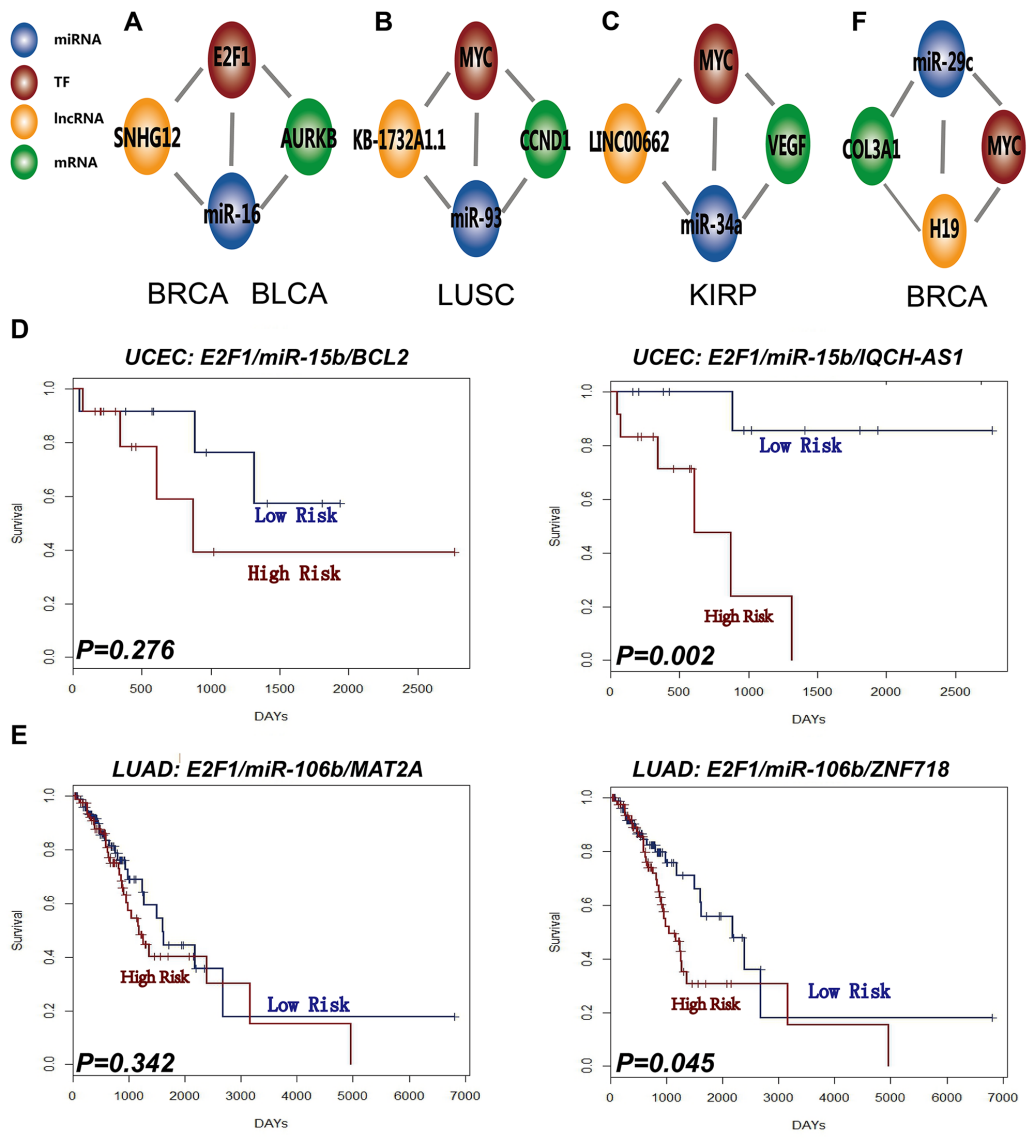
L-FFL motifs participate in complex biology network regulation An example of network regulation when combining the dysregulated M-FFL and L-FFL motif in **A.** BRCA and BLCA, **B.** LUSC, and **C.** KIRP. **D.** Survival analysis of the M-FFL motif (left) and L-FFL motif (right) in UCEC, and **E.** LUAD. **F**. Example of network regulation when combining dysregulated ceRNA and L-FFL motif in BRCA.

We investigated whether there is cross-talk between the L-FFL motif and other types of network motifs in human cancer. First, we identified dysregulated mRNA-mediated FFL (M-FFL) motifs in different types of cancers by following the same pipeline as with the L-FFL motif identification. Then, we generated dysregulated motifs that included the TFs and miRNAs shared by both the L-FFL and the M-FFL motifs (L-M-FFL motif). For example, we found that an L-FFL motif constituted by SNHG12, E2F1, and miR-16, and an M-FFL motif constituted by E2F1, miR-16 and AURKB could form a complex regulation motif by sharing common TF E2F1 and miRNA miR-16, which were highly dysregulated in breast and bladder cancers (Figure 5A, Supplementary Table S6). In addition, some L-M-FFL motifs were dysregulated in only one type of cancer and could be regarded as cancer-specific motifs. For example, an L-M-FFL motif comprised of KB-1732A1.1, MYC, miR-93, and CCND1 was only dysregulated in lung squamous cell carcinomas (Figure 5B), and an L-M-FFL motif including LINC00662, MYC, miR-34a and VEGF was only dysregulated in kidney cancer (Figure 5C). Interestingly, we found cases of L-M-FFL motifs for which only the L-FFL motif exhibited functional relevance. For example, an L-FFL motif constituted by E2F1, miR-15b, and IQCH-AS1, and an M-FFL motif made of E2F1, miR-15b and BCL2 formed a complex L-M-FFL motif in uterine corpus endometrioid carcinoma, but only the L-FFL motif correlated with patient survival (P=0.002, Figure 5D). The same result was also found in lung adenocarcinoma cancer (Figure 5E). These results indicate that although different types of motifs participate in cross-talk within the network, only parts of the network motif have a specific function.

We also obtained ceRNA motifs associated with cancer from the LncACTdb database [16]. We found that some dysregulated L-FFL and ceRNA motifs shared common miRNAs and lncRNAs in some types of cancers. For example, an L-FFL motif comprised of the lncRNA H19, the TF MYC and the miRNA miR-29c, and an ceRNA motif comprised of the mRNA COL3A1, the lncRNA H19 and the miRNA miR-29c, were both dysregulated in breast cancer (Figure 5F). In this case, the L-FFL and ceRNA motifs might exhibit complex regulatory functions and cross-talk in cancer.

## DISCUSSION

**Figure 6:**
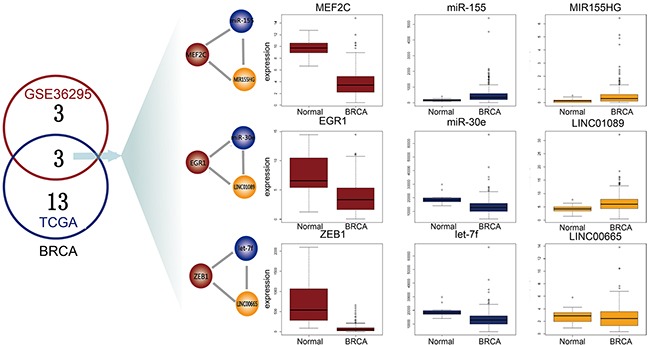
Common L-FFL motifs between microarray and RNA-seq data The numbers of dysregulated L-FFL motifs in GEO and TCGA samples for BRCA are shown in red and blue circles, respectively. Three common dysregulated L-FFL motifs are shown. The box plots show the differential expression of each L-FFL motif, and TFs, miRNAs and lncRNAs are colored red, blue and yellow, respectively.

The cross-talk between different types of regulatory transcripts (e.g., mRNAs, lncRNAs, TFs and miRNAs) forms complex network motifs that may underlie carcinogenesis in some human cancers. In this study, we focused on a novel network motif, the L-FFL motif, and developed a computational approach to study it by integrating interaction and expression data of lncRNA, mRNAs and miRNAs. To validate our approach, we performed the same analyses using an independent microarray dataset from Gene Expression Omnibus (GEO, GSE36295) that included 45 breast cancer samples and eight normal samples. We obtained the TF, miRNA and lncRNA expression data for these samples based on the re-annotation of the Affymetrix Human Gene 1.0 ST Array and identified six dysregulated L-FFL motifs. There were three common dysregulated L-FFL motifs between GEO microarray and TCGA RNA-seq data, and TFs, miRNAs and lncRNAs were differentially expressed between breast cancer and normal samples (Figure 6).

In this study, we also identified dysregulated L-FFL motifs that were common and specific to several cancers, consistent with previous studies on the tissue specificity of miRNA and lncRNA [45]. Tissue-specific L-FFL motifs could aid the development of drugs that target specific cancer tissues while minimizing side effects. Our functional and survival analyses suggest that L-FFL motifs may serve as prognostic biomarkers for cancer. Similar to previous studies highlighting the use of lncRNAs as cancer biomarkers [46], we found that the expression of lncRNAs that participate in L-FFL motifs correlates with prognosis.

There are other types of L-FFL motifs different from the ones we focused on in this study, such as those in which miRNAs and lncRNAs regulate TFs or lncRNAs regulate miRNAs [47]. Under different mechanisms and conditions, TFs can activate or repress the expression of target lncRNAs and miRNAs in the L-FFL motifs [21]. These alternate L-FFL motifs have also been shown to participate in the development of several types of cancer [48, 49], and can also be constructed with the help of databases [50]. Further analyses of these types of motifs and their complex regulatory patterns could also reveal novel mechanism underlying carcinogenesis.

In summary, our study provides novel insights into the mechanisms underlying the function of lncRNAs in cancer. As shown here, the analysis of the L-FFL network can help to understand the relevance of multi-level cross-talk regulation in cancer by revealing functional motifs that are common across or specific to different cancer types.

## MATERIALS AND METHODS

### Expression data of lncRNA, TF and miRNA

We obtained the data from six types of cancers and normal expression data of lncRNAs, TFs and miRNAs as previously described [16]. Briefly, we downloaded the raw read counts for each exon from the TCGA level 3 dataset. Then, we mapped these exons to the annotation of human TFs and lncRNAs that was derived from GENCODE [51]. We recalculated the reads per kilobases per million reads (RPKM) values for the TFs and lncRNAs, leading to expression data of human TF and lncRNA. The miRNA sequencing data (Illumina HiSeq miRNASeq) for six types of cancer were downloaded from TCGA (level 3) [52]. Cancer samples with clinical follow-up information were retained for further analysis. The cancer name abbreviations of TCGA and the number of cancer and normal samples are listed in Supplementary Table S7.

### Constructing a global L-FFL network

L-FFL motifs consist of a TF, an miRNA and their common target lncRNA, in which the TF regulates the expression of the miRNA, and both the TF and the miRNA regulate a common set of target lncRNAs [7]. To construct a global L-FFL network, three types of regulatory interaction are needed: miRNA-lncRNA, TF-lncRNA and TF-miRNA. The regulatory interactions chosen in this study were downloaded from starBase v2.0 [20], which provides comprehensive CLIP-Seq experimentally supported miRNA-lncRNA interaction data. The TF-lncRNA regulatory interactions were downloaded from SNP@lincTFBS [18], which identifies transcription factor binding sites (TFBSs) of lncRNA using genome-wide ChIP-Seq data. Finally, the TF-miRNA regulatory interactions were downloaded from TransmiR [21], which manually collects experimentally supported TF-miRNA regulatory relationships from literature and publications.

### Topological measurements of the L-FFL network

We investigated several common topological measurements to reveal the characteristics of the L-FFL network. For the whole L-FFL network, we analyzed the degrees, connectivity, topological coefficients, and clustering coefficients of nodes (lncRNA, TF and miRNA).

### Identifying L-FFLs dysregulated in cancer

Using both the L-FFL network and expression profiling data, we developed an integrative pipeline to detect L-FFL motifs dysregulated in different types of cancer (Supplementary Figure S2). First, for each L-FFL motif, we computed t-tests or paired t-tests and P-values to measure the differences in the expression levels of lncRNAs, TFs and miRNAs between cancer and normal samples. For each miRNA-lncRNA, TF-lncRNA and TF-miRNA regulatory interaction in each L-FFL motif, we calculated Pearson correlation coefficients (PCCs) for the cancer and normal samples, as well as the difference between them. We used the absolute difference between cancer and normal PCCs to represent association between interactions. We integrated the differential expression P-value and PCCs to calculate two comprehensive scores (score_diff_ and score_cor_) for L-FFL as follows (Supplementary Figure S3):

$$\begin{array}{l} {\text{score}}_{\text{diff}}=p_{t}p_{m}p_{1}\\ {\text{score}}_{\text{cor}}=|(C_{tm}-N_{tm})(C_{tl}-N_{tl})(C_{ml}-N_{ml})| \end{array}$$

where *p_t_*, p*_m_*, and *p_l_* are the differential expression P-values of TF, miRNA and lncRNA, respectively, in each L-FFL, and *score_diff_* corresponds to the difference in expression of an L-FFL motif between the cancer and normal samples. *C_tm_*, C*_tl_,* and *C_ml_* are the PCCs for the TF and miRNA, TF and lncRNA, and miRNA and lncRNA pairs, respectively, in the cancer samples, while *N_tm_*, N*_tl_,* and *N_ml_* are the PCCs for the TF and miRNA, TF and lncRNA, and miRNA and lncRNA pairs, respectively, in the normal samples. *Score_cor_* corresponds to the absolute difference in the correlation level of an entire L-FFL motif between the cancer and normal samples. Second, we ranked all of the L-FFL motifs for each cancer type using *score_diff_* and *score_cor_* based on an equally-weighted, multidimensional ranking method [53]. After ranking by each score, we computed a final ranking score for each L-FFL motif by integrating its two rank positions in a two-rank list. A higher ranking corresponds to larger dysregulation in cancer. Third, we measured the significance of each L-FFL motif's ranking by comparing its final score with that of L-FFL motifs calculated by randomly permuting the sample labels for expression profiling. With 1000 permutations, we referred to previous studies and generated dysregulated L-FFL motifs for each type of cancer based on the permutation P-value (P < 0.05)[47].

### Survival analysis

For each dysregulated L-FFL motif in each type of cancer, a Cox multiple regression analysis was used to evaluate the association between expression and cancer survival. The corresponding regression coefficient of each lncRNA, miRNA and TF in an L-FFL motif was used as a weighting coefficient to produce an integrated score. We used the integrated score to classify the samples into high-risk and low-risk groups. Then, a Kaplan-Meier survival analysis was performed for the two clustered groups, and statistical significance was assessed using the log-rank test. All of the analyses were performed within the R 2.15.3 framework.

### Gene set enrichment analysis

Gene Ontology (GO) enrichment and KEGG pathways enrichment analyses were performed by the DAVID functional annotation web server using default parameters [40]. We obtained enriched GO terms (FDR < 0.05) and KEGG pathways (P < 0.1).

## SUPPLEMENTARY FIGURES AND TABLES









## References

[1] Bassett AR, Akhtar A, Barlow DP, Bird AP, Brockdorff N, Duboule D, Ephrussi A, Ferguson-Smith AC, Gingeras TR, Haerty W, Higgs DR, Miska EA, Ponting CP (2014). Considerations when investigating lncRNA function in vivo. Elife.

[2] St Laurent G, Wahlestedt C, Kapranov P (2015). The Landscape of long noncoding RNA classification. Trends Genet.

[3] Quinn JJ, Chang HY (2015). Unique features of long non-coding RNA biogenesis and function. Nat Rev Genet.

[4] Zhang M, Gu H, Xu W, Zhou X (2016). Down-regulation of lncRNA MALAT1 reduces cardiomyocyte apoptosis and improves left ventricular function in diabetic rats. Int J Cardiol.

[5] Huang W, Thomas B, Flynn RA, Gavzy SJ, Wu L, Kim SV, Hall JA, Miraldi ER, Ng CP, Rigo FW, Meadows S, Montoya NR, Herrera NG (2015). DDX5 and its associated lncRNA Rmrp modulate TH17 cell effector functions. Nature.

[6] Gupta RA, Shah N, Wang KC, Kim J, Horlings HM, Wong DJ, Tsai MC, Hung T, Argani P, Rinn JL, Wang Y, Brzoska P, Kong B (2010). Long non-coding RNA HOTAIR reprograms chromatin state to promote cancer metastasis. Nature.

[7] Salmena L, Poliseno L, Tay Y, Kats L, Pandolfi PP (2011). A ceRNA hypothesis: the Rosetta Stone of a hidden RNA language?. Cell.

[8] Guttman M, Rinn JL (2012). Modular regulatory principles of large non-coding RNAs. Nature.

[9] Redmond AM, Carroll JS (2009). Defining and targeting transcription factors in cancer. Genome Biol.

[10] Shields BB, Pecot CV, Gao H, McMillan E, Potts M, Nagel C, Purinton S, Wang Y, Ivan C, Kim HS, Borkowski RJ, Khan S, Rodriguez-Aguayo C (2015). A genome-scale screen reveals context-dependent ovarian cancer sensitivity to miRNA overexpression. Mol Syst Biol.

[11] Legnini I, Morlando M, Mangiavacchi A, Fatica A, Bozzoni I (2014). A feedforward regulatory loop between HuR and the long noncoding RNA linc-MD1 controls early phases of myogenesis. Mol Cell.

[12] Yan Z, Shah PK, Amin SB, Samur MK, Huang N, Wang X, Misra V, Ji H, Gabuzda D, Li C (2012). Integrative analysis of gene and miRNA expression profiles with transcription factor-miRNA feed-forward loops identifies regulators in human cancers. Nucleic Acids Res.

[13] Li M, Izpisua Belmonte JC (2015). Roles for noncoding RNAs in cell-fate determination and regeneration. Nat Struct Mol Biol.

[14] Chen CL, Tseng YW, Wu JC, Chen GY, Lin KC, Hwang SM, Hu YC (2015). Suppression of hepatocellular carcinoma by baculovirus-mediated expression of long non-coding RNA PTENP1 and MicroRNA regulation. Biomaterials.

[15] Yan X, Hu Z, Feng Y, Hu X, Yuan J, Zhao SD, Zhang Y, Yang L, Shan W, He Q, Fan L, Kandalaft LE, Tanyi JL (2015). Comprehensive Genomic Characterization of Long Non-coding RNAs across Human Cancers. Cancer Cell.

[16] Wang P, Ning S, Zhang Y, Li R, Ye J, Zhao Z, Zhi H, Wang T, Guo Z, Li X (2015). Identification of lncRNA-associated competing triplets reveals global patterns and prognostic markers for cancer. Nucleic Acids Res.

[17] Yang JH, Li JH, Jiang S, Zhou H, Qu LH (2013). ChIPBase: a database for decoding the transcriptional regulation of long non-coding RNA and microRNA genes from ChIP-Seq data. Nucleic Acids Res.

[18] Ning S, Zhao Z, Ye J, Wang P, Zhi H, Li R, Wang T, Wang J, Wang L, Li X (2014). SNP@lincTFBS: an integrated database of polymorphisms in human LincRNA transcription factor binding sites. PLoS One.

[19] Paraskevopoulou MD, Georgakilas G, Kostoulas N, Reczko M, Maragkakis M, Dalamagas TM, Hatzigeorgiou AG (2013). DIANA-LncBase: experimentally verified and computationally predicted microRNA targets on long non-coding RNAs. Nucleic Acids Res.

[20] Li JH, Liu S, Zhou H, Qu LH, Yang JH (2014). starBase v2. 0: decoding miRNA-ceRNA, miRNA-ncRNA and protein-RNA interaction networks from large-scale CLIP-Seq data. Nucleic Acids Res.

[21] Wang J, Lu M, Qiu C, Cui Q (2010). TransmiR: a transcription factor-microRNA regulation database. Nucleic Acids Res.

[22] Amaral LA, Scala A, Barthelemy M, Stanley HE (2000). Classes of small-world networks. Proc Natl Acad Sci U S A.

[23] Simos T, Georgopoulou U, Thyphronitis G, Koskinas J, Papaloukas C (2015). Analysis of protein interaction networks for the detection of candidate hepatitis B and C biomarkers. IEEE J Biomed Health Inform.

[24] Xu J, Li Y, Lu J, Pan T, Ding N, Wang Z, Shao T, Zhang J, Wang L, Li X (2015). The mRNA related ceRNA-ceRNA landscape and significance across 20 major cancer types. Nucleic Acids Res.

[25] Chen G, Wang Z, Wang D, Qiu C, Liu M, Chen X, Zhang Q, Yan G, Cui Q (2013). LncRNADisease: a database for long-non-coding RNA-associated diseases. Nucleic Acids Res.

[26] Li Y, Qiu C, Tu J, Geng B, Yang J, Jiang T, Cui Q (2014). HMDD v2.0: a database for experimentally supported human microRNA and disease associations. Nucleic Acids Res.

[27] Mathsyaraja H, Eisenman RN (2016). Parsing Myc Paralogs in Oncogenesis. Cancer Cell.

[28] Huang X, Yang Y, Guo Y, Cao ZL, Cui ZW, Hu TC, Gao LB (2015). Association of a let-7 KRAS rs712 polymorphism with the risk of breast cancer. Genet Mol Res.

[29] Zhang E, Li W, Yin D, De W, Sun S, Han L (2015). c-Myc-regulated long non-coding RNA H19 indicates a poor prognosis and affects cell proliferation in non-small-cell lung cancer. Tumour Biol.

[30] Tan M, Wu J, Cai Y (2013). Suppression of Wnt signaling by the miR-29 family is mediated by demethylation of WIF-1 in non-small-cell lung cancer. Biochem Biophys Res Commun.

[31] Boeri M, Verri C, Conte D, Roz L, Modena P, Facchinetti F, Calabro E, Croce CM, Pastorino U, Sozzi G (2011). MicroRNA signatures in tissues and plasma predict development and prognosis of computed tomography detected lung cancer. Proc Natl Acad Sci U S A.

[32] Xie C, Yuan J, Li H, Li M, Zhao G, Bu D, Zhu W, Wu W, Chen R, Zhao Y (2014). NONCODEv4: exploring the world of long non-coding RNA genes. Nucleic Acids Res.

[33] Salvador MA, Wicinski J, Cabaud O, Toiron Y, Finetti P, Josselin E, Lelievre H, Kraus-Berthier L, Depil S, Bertucci F, Collette Y, Birnbaum D, Charafe-Jauffret E, Ginestier C (2013). The histone deacetylase inhibitor abexinostat induces cancer stem cells differentiation in breast cancer with low Xist expression. Clin Cancer Res.

[34] Ostrander JH, Daniel AR, Lofgren K, Kleer CG, Lange CA (2007). Breast tumor kinase (protein tyrosine kinase 6) regulates heregulin-induced activation of ERK5 and p38 MAP kinases in breast cancer cells. Cancer Res.

[35] Gotte M, Mohr C, Koo CY, Stock C, Vaske AK, Viola M, Ibrahim SA, Peddibhotla S, Teng YH, Low JY, Ebnet K, Kiesel L, Yip GW (2010). miR-145-dependent targeting of junctional adhesion molecule A and modulation of fascin expression are associated with reduced breast cancer cell motility and invasiveness. Oncogene.

[36] Schrauder MG, Strick R, Schulz-Wendtland R, Strissel PL, Kahmann L, Loehberg CR, Lux MP, Jud SM, Hartmann A, Hein A, Bayer CM, Bani MR, Richter S (2012). Circulating micro-RNAs as potential blood-based markers for early stage breast cancer detection. PLoS One.

[37] Du L, Schageman JJ, Subauste MC, Saber B, Hammond SM, Prudkin L, Wistuba II, Ji L, Roth JA, Minna JD, Pertsemlidis A (2009). miR-93, miR-98, and miR-197 regulate expression of tumor suppressor gene FUS1. Mol Cancer Res.

[38] Lv J, Yu YQ, Li SQ, Luo L, Wang Q (2014). Aflatoxin B1 promotes cell growth and invasion in hepatocellular carcinoma HepG2 cells through H19 and E2F1. Asian Pac J Cancer Prev.

[39] Berteaux N, Lottin S, Monte D, Pinte S, Quatannens B, Coll J, Hondermarck H, Curgy JJ, Dugimont T, Adriaenssens E (2005). H19 mRNA-like noncoding RNA promotes breast cancer cell proliferation through positive control by E2F1. J Biol Chem.

[40] Huang da W, Sherman BT, Lempicki RA (2009). Systematic and integrative analysis of large gene lists using DAVID bioinformatics resources. Nat Protoc.

[41] Tarangelo A, Lo N, Teng R, Kim E, Le L, Watson D, Furth EE, Raman P, Ehmer U, Viatour P (2015). Recruitment of Pontin/Reptin by E2f1 amplifies E2f transcriptional response during cancer progression. Nat Commun.

[42] Guo CJ, Pan Q, Li DG, Sun H, Liu BW (2009). miR-15b and miR-16 are implicated in activation of the rat hepatic stellate cell: An essential role for apoptosis. J Hepatol.

[43] Barrans S, Crouch S, Smith A, Turner K, Owen R, Patmore R, Roman E, Jack A (2010). Rearrangement of MYC is associated with poor prognosis in patients with diffuse large B-cell lymphoma treated in the era of rituximab. J Clin Oncol.

[44] Landi MT, Zhao Y, Rotunno M, Koshiol J, Liu H, Bergen AW, Rubagotti M, Goldstein AM, Linnoila I, Marincola FM, Tucker MA, Bertazzi PA, Pesatori AC, Caporaso NE, McShane LM, Wang E (2010). MicroRNA expression differentiates histology and predicts survival of lung cancer. Clin Cancer Res.

[45] Grote P, Wittler L, Hendrix D, Koch F, Wahrisch S, Beisaw A, Macura K, Blass G, Kellis M, Werber M, Herrmann BG (2013). The tissue-specific lncRNA Fendrr is an essential regulator of heart and body wall development in the mouse. Dev Cell.

[46] Li J, Chen Z, Tian L, Zhou C, He MY, Gao Y, Wang S, Zhou F, Shi S, Feng X, Sun N, Liu Z, Skogerboe G (2014). LncRNA profile study reveals a three-lncRNA signature associated with the survival of patients with oesophageal squamous cell carcinoma. Gut.

[47] Jiang W, Mitra R, Lin CC, Wang Q, Cheng F, Zhao Z (2015). Systematic dissection of dysregulated transcription factor-miRNA feed-forward loops across tumor types. Brief Bioinform.

[48] Fang Z, Xu C, Li Y, Cai X, Ren S, Liu H, Wang Y, Wang F, Chen R, Qu M, Wang Y, Zhu Y, Zhang W (2016). A feed-forward regulatory loop between androgen receptor and PlncRNA-1 promotes prostate cancer progression. Cancer Lett.

[49] Liu X, Xiao ZD, Han L, Zhang J, Lee SW, Wang W, Lee H, Zhuang L, Chen J, Lin HK, Wang J, Liang H, Gan B (2016). LncRNA NBR2 engages a metabolic checkpoint by regulating AMPK under energy stress. Nat Cell Biol.

[50] Li J, Ma W, Zeng P, Wang J, Geng B, Yang J, Cui Q (2015). LncTar: a tool for predicting the RNA targets of long noncoding RNAs. Brief Bioinform.

[51] Kersey PJ, Lawson D, Birney E, Derwent PS, Haimel M, Herrero J, Keenan S, Kerhornou A, Koscielny G, Kahari A, Kinsella RJ, Kulesha E, Maheswari U (2010). Ensembl Genomes: extending Ensembl across the taxonomic space. Nucleic Acids Res.

[52] Cancer Genome Atlas Research N (2012). Comprehensive genomic characterization of squamous cell lung cancers. Nature.

[53] Aerts S, Lambrechts D, Maity S, Van Loo P, Coessens B, De Smet F, Tranchevent LC, De Moor B, Marynen P, Hassan B, Carmeliet P, Moreau Y (2006). Gene prioritization through genomic data fusion. Nat Biotechnol.

