# Supplementary material for: Construction of a lncRNA-mediated feed-forward loop network reveals global topological features and prognostic motifs in human cancers
Source: Oncotarget. 2016 Jun 14;7(29):45937–47. doi: 10.18632/oncotarget.10004 (PMC5216772; doi:10.18632/oncotarget.10004)
Supplement: Supplementary file 1 [file oncotarget-07-45937-s001.pdf]

Construction of a lncRNA-mediated feed-forward loop network reveals global topological features and prognostic motifs in human cancers

SUPPLEMENTARY FIGURES AND TABLES

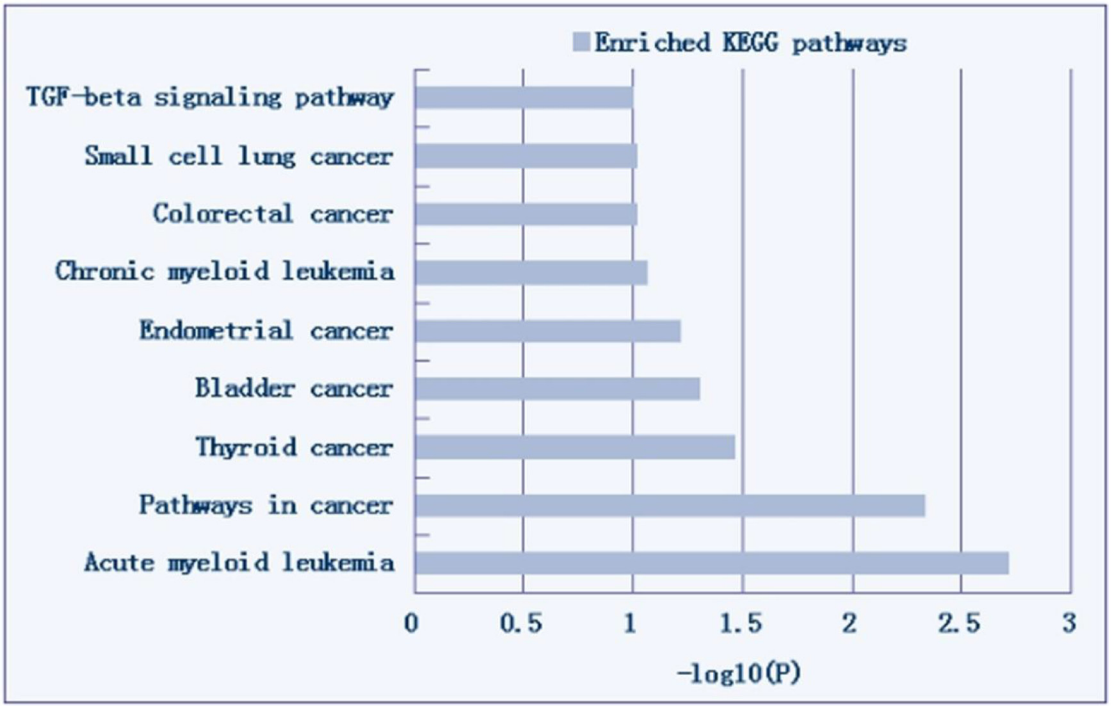

Supplementary Figure S1: KEGG analysis with TFs in significantly dysregulated L-FFL motifs. The length of the bar chart represents the P-value of the enriched KEGG pathway.

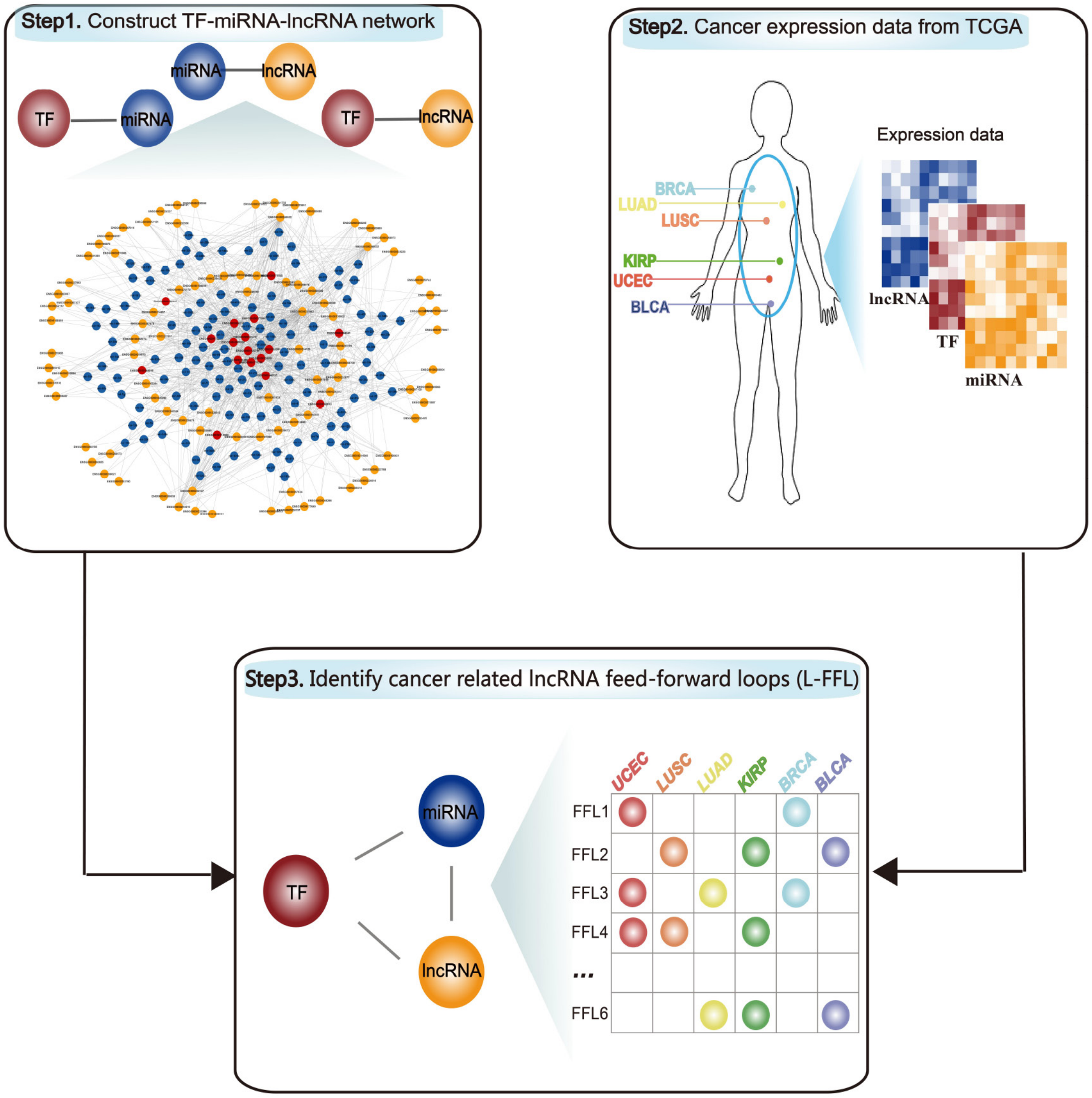

Supplementary Figure S2: The workflow of our study. We constructed an L-FFL network using different data sources (Step 1), obtained lncRNA, TF and miRNA expression profiles from the TCGA datasets (Step 2), and identified significantly dysregulated L-FFL motifs through an integration of network motifs and expression data (Step 3).

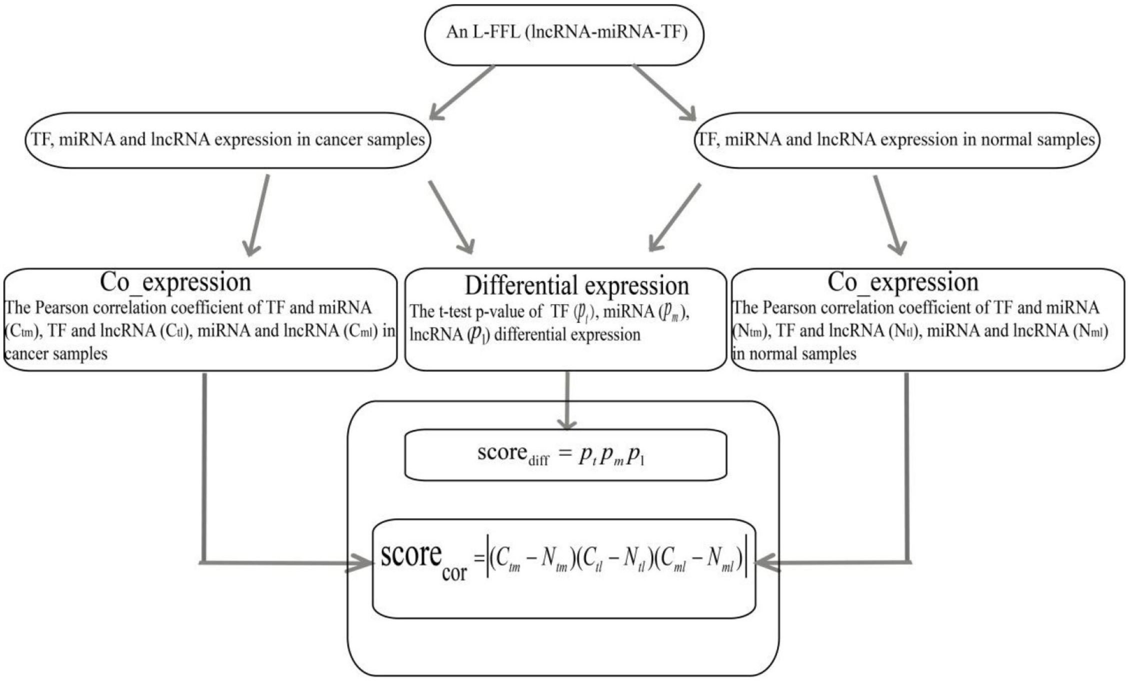

Supplementary Figure S3: The workflow of identifying significantly dysregulated L-FFL motifs for cancer.

Supplementary Table S1: Known disease-associated lncRNAs, TFs and miRNAs in the L-FFL network.

See Supplementary File 1

Supplementary Table S2: Significantly dysregulated L-FFL motifs in each type of cancer.

See Supplementary File 2

Supplementary Table S3: Significantly enriched GO terms in dysregulated L-FFL motifs.

See Supplementary File 3

Supplementary Table S4: Common and specific dysregulated L-FFL motifs across cancer types

LncRNA	TF	miRNA	Cancer	P value
IQCH-AS1	E2F1	miR-15b	UCEC,LUSC,BLCA	0.05,0.002,0.034
SNHG12	E2F1	miR-195	LUAD,KIRP,BLCA	0.036,0.022,0.036
KB-1732A1.1	E2F1	miR-106b	LUSC,LUAD,BRCA	0.029,0.032,0.005
LINC00665	MYC	miR-98	BRCA	0.021
JPX	MEF2C	miR-145	BLCA	0.016
MAPKAPK5-AS1	MYC	miR-429	KIRP	0.048
ZNF718	E2F1	miR-106b	LUAD	0.012
EPB41L4A-AS1	E2F1	miR-16	UCEC	0.022
H19	MYC	miR-93	LUSC	0.007

Supplementary Table S5: L-FFL motifs as prognostic biomarkers for cancers

LncRNA	TF	miRNA	Cancer	P value
JPX	MEF2C	miR-145	BLCA	0.026
LINC00665	MYC	miR-98	BRCA	0.023
MAPKAPK5-AS1	MYC	miR-429	KIRP	0.065
ZNF718	E2F1	miR-106b	LUAD	0.045
EPB41L4A-AS1	E2F1	miR-16	UCEC	0.019
IQCH-AS1	E2F1	miR-15b	UCEC	0.002
miR155HG	MEF2C	miR-194	BRCA	0.010
IQCH-AS1	E2F1	miR-15a	UCEC	0.017
EPB41L4A-AS1	TCF7L2	miR-214	UCEC	0.016

Supplementary Table S6: L-FFL motifs may participate in complex biologic network regulation

LncRNA	TF	miRNA	mRNA	L-FFL P value	M-FFL P value	Cancer
SNHG12	E2F1	miR-16	AURKB	0.012,0.014	0.012,0.012	BRCA,BLCA
KB-173A1.1	MYC	miR-93	CCND1	0.007	0.049	LUSC
lINC00662	MYC	miR-34a	VEGF	0.032	0.014	KIRP
H19	MYC	miR-29c	COL3A1	0.04	0.03	BRCA

Supplementary Table S7: Cancer name abbreviations of TCGA and cancer and normal samples

Abbreviations	Cancer names	Cancer samples	Normal samples
BLCA	Bladder Urothelial Carcinoma	81	16
BRCA	Breast invasive carcinoma	248	15
KIRP	Kidney renal papillary cell carcinoma	67	9
LUAD	Lung adenocarcinoma	166	9
LUSC	Lung squamous cell carcinoma	120	9
UCEC	Uterine Corpus Endometrioid Carcinoma	24	6
